# Value of Proton-MR-Spectroscopy in the Diagnosis of Temporal Lobe Epilepsy; Correlation of Metabolite Alterations With Electroencephalography

**DOI:** 10.5812/iranjradiol.6686

**Published:** 2012-03-25

**Authors:** Hasan Aydin, Nilay Aydin Oktay, Volkan Kizilgoz, Elif Altin, Idil Gunes Tatar, Baki Hekimoglu

**Affiliations:** 1 MRI Department, S.B. Ankara Diskapi Yildirim Beyazit Education and Research Hospital, Ankara, Turkey; 2 Vascular Interventional Department, S.B. Ankara Diskapi Yildirim Beyazit Education and Research Hospital, Ankara, Turkey

**Keywords:** Magnetic Resonance Spectroscopy, Epilepsy, Temporal Lobe, Electroencephalography

## Abstract

**Background:**

Epilepsy, a well-known mostly idiopathic neurologic disorder, has to be correctly diagnosed and properly treated. Up to now, several diagnostic approaches have been processed to determine the epileptic focus.

**Objectives:**

The aim of this study was to discover whether proton-MR-spectroscopic imaging (MRSI) aids in the diagnosis of temporal lobe epilepsy in conjunction with classical electroencephalography (EEG) findings.

**Patients and Methods:**

Totally, 70 mesial temporal zones consisting of 39 right hippocampi and 31 left hippocampi of 46 patients (25 male, 21 female) were analyzed by proton MRSI. All patients underwent a clinical neurologic examination, scalp EEG recording and prolonged video EEG monitoring. Partial seizures on the right, left or both sides were recorded in all patients. All patients were under medical treatment and none of the patients underwent amygdalohippocampectomy and similar surgical procedures.

**Results:**

The normal average lactate (Lac), phosphocreatine, N-acetyl aspartate (NAA), creatine (Cr), choline (Cho), myo-inositol, glutamate and glutamine (Glx) peaks and Nacetyl aspartate/Cr, NAA/ Cho + Cr, Cho/Cr ratios were measured from the healthy opposite hippocampi or from the control subjects. The Lac, glutamate and glutamine (Glx), myo-inositol, phosphocreatine and NAA metabolites plus Cho/Cr ratio showed statistical difference between the normal and the epileptic hippocampi. Cho, Cr metabolites plus NAA/Cr, NAA/ Cho + Cr ratios were almost the same between the groups. The sensitivity of Proton-MR-Spectroscopy for lateralization of the epileptic foci in all patients was 96% and the specificity was 50%.

**Conclusions:**

Proton-MRSI can easily be considered as an alternative modality of choice in the diagnosis of temporal lobe epilepsy and in the future; Proton-MR-Spectroscopy may become the most important technique used in epilepsy centers.

## 1. Background

Epilepsy is a group of neurological abnormalities in which the most common and fundamental characteristic is recurrent and mostly unprovoked seizures [1]. The subgroups are idiopathic epilepsies which occur in a structurally normal brain and have a presumably genetic cause and symptomatic epilepsies which are caused by a known structural, focal or diffuse abnormality [1][2]. The most common class of symptomatic epilepsies is the mesial temporal lobe epilepsy (MTLE) frequently associated with hippocampal sclerosis [1][2][3][4]. The most important tools in the diagnosis of MTLE are the clinical neurological features and the intensive video-EEG monitoring [1][3][4][5][6]. Neuropsychological evaluation, brain magnetic resonance imaging (MRI), positron emission tomography (PET) and single photon emission computed tomography (SPECT) imaging are the other diagnostic approaches in the assessment of epileptic foci [3][4][5][6][7]. Cerebral MRI usually shows atrophy or high signal intensity in the affected hippocampus or amygdala; however, in some patients with MTLE, it may reveal no abnormalities [1][3][7][8][9]. Proton-MR-Spectroscopy (H-MRS), a non-invasive technique, has been suggested for its usefulness in the evaluation of MTLE and epileptogenic areas. By detection of metabolites in the mesial temporal structures (hippocampus and the amygdala), H-MRS can give sensitive and reliable results, may aid in the lateralization of epileptic foci and the diagnosis of MTLE [1][3][4][6][7][8][9][10][11]. Lateralization is a procedure that is used to determine the hemisphere which is responsible for the genesis of seizures [1][4][6][7][11].

## 2. Objectives

In this study, the aim was to discover the potential benefits of H-MRS in the lateralization of epileptic foci in the mesial temporal parts, mainly the hippocampi, correlated with the EEG findings and the clinical features.

## 3. Patients and Methods

Forty-six consecutive patients (25 male-21 female) in the age range of 11-62 years (mean, 35 ± 4 years) were included in this study. Thirty-nine right hippocampi and 31 left hippocampi, totally 70 mesial temporal zones were analyzed in these patients. Twenty-two of the patients had bilateral and 24 had unilateral hippocampal involvement. All patients were evaluated by the neurology department of our hospital. In the MRI, they had no neoplastic or traumatic lesions. Only one patient had a subacute hematoma in the left temporal lobe. All patients underwent a clinical neurologic examination, scalp EEG recording and prolonged video EEG monitoring. Complex partial seizures on the right, left or both sides were recorded in all patients and none of the patients had a second seizure focus. All patients were under medical treatment and none of the patients underwent amygdalohippocampectomy and similar surgical procedures. Follow up of the patients and duration of epilepsy were about 2 months to 26 years. In this group; there were chronic epileptics-late epilepsy patients and also patients with acute onset seizures. All the patients and the healthy control group participated in this study with their own consent.

All the MRIs and spectroscopic analysis were carried out via 1.5 T (Philips Achieva, 8 Channel, Philips Medical System, Netherlands) scanner using a standard head coil. Routine brain MRI was performed using 3D-Flair, T2 weighted fast field echo (FFE) and T2 weighted turbo spin echo (TSE) coronal sequences. Axial T1 and T2 weighted TSE sequences were also applied. Multi-voxel spectroscopic imaging (MV-MRS) was performed using point-resolved spectroscopy (PRESS) with a standard volume of interest (VOI) size, 0.5 × 0.5 × 1 cm for all hippocampi. We positioned the voxel over the mesial temporal lobe to cover most of the hippocampus and tried to minimize partial-volume effects resulting from other neighbouring tissues including amygdala, cerebral spinal fluid (CSF) of the ventricles and parahippocampal gyrus. The voxel mostly comprised the head and anterior part of the hippocampi. Time domain data were multiplied by a Gaussian function of 90 (Center 0, halfwidth 256 ms), 2D Fourier transformed phase and base-line corrected, quantified by means of frequency domain curve fitting with the assumption of a Gaussian line shape using Philips software. A 0-4.35 ppm was analyzed and metabolite signal peaks were centered as follows; N-acetyl aspartate (NAA) at 2 ppm, creatine (Cr) at 3-3.1 ppm, phosphocreatine (Cr2) at 3.8-3.9 ppm, choline (Cho) at 3.2 ppm, lactate (Lac) at 1.3-1.4 ppm, glutamate and glutamine (Glx) at 2.45 ppm, glycine and myo-inositol (Gly-MI) at 3.6-3.75 ppm ([1], [7], [10]-[12]). Detailed Glx-MI-Cr2 metabolite concentrations were briefly presented in TE: 26 ms and NAA-Cho-Cr-Lac metabolites were mostly analyzed in TE: 144 ms. Automatic shimming of the linear x, y, z channels was used to optimize field homogeneity, water resonance and water suppression pulses were optimized. Proton spectra were recorded in the coronal plane with T2 weighted images via TR; 1500 ms, TE; 26 and 144 ms, FOV; 24 × 24 cm, 1-1.5 cm section thickness, 256 × 256 matrix and 24 × 24 phase encoding. Spectral analysis and last processing were carried out using Philips software (Philips Achieva, 8 Channel, Netherlands) work-shop. All the MRSI data and metabolite concentrations were evaluated by two radiologists, 3 and 7 years experienced, together with consensus so there was no intra- and interobserver variability.

Lateralization of epileptic foci using MRSI was performed in two ways; the first lateralization involved comparison of metabolite concentrations and metabolite peak ratios in the ipsilateral hippocampal region with those on the opposite side, this was applied when there was unilateral involvement in EEG. When there was bilateral involvement in EEG, we compared the hippocampal data of patients upon the mesial temporal zone data of five healthy control subjects. The control group was selected from non-epileptic patients with their own willingness to participate in this research who were referred to our unit for cranial MRI due to headache, dizziness, vertigo and similar symptoms. These five healthy controls had normal cerebral and cerebellar MRI findings. Values more than 2 standard deviations below the mean data of control subjects were considered abnormal. The control group was composed of healthy subjects with an unknown seizure history and no EEG abnormality, aged between 20 and 45 years (mean, 32 ± 3 years).

All statistical analyses were performed using a software program, Statistical Package for the Social Sciences (SPSS for Windows ver. 15.0, Chicago-Illinois). Measurements of metabolite peaks, their alterations and metabolite ratios were analyzed by paired t-test for both hippocampal involvement and single-sampling t-test for all patients including bilateral and unilateral involvement. P < 0.05 was considered to be statistically significant. Receiver operating characteristic (ROC) curve analysis for metabolite peaks, ratios and cut-off points with regard to area under curve (AUC) were also determined.

## 4. Results

The normal average Lac-Cr2-NAA-Cre-Cho-MI-Glx peaks and NAA/Cr, NAA/Cho + Cr, Cho/Cr ratios were measured in all patients and also in the healthy opposite hippocampi or in the control subjects.

**Figure 1:**
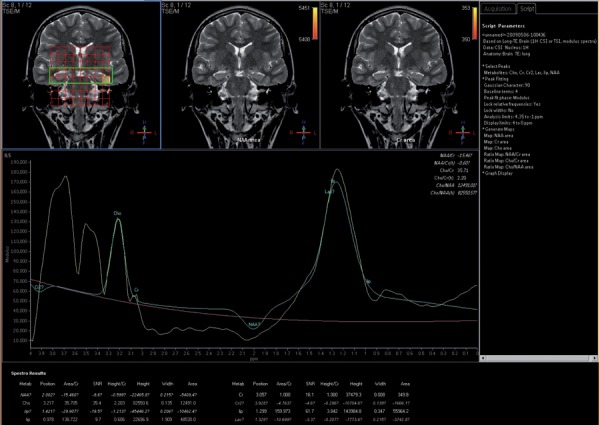
A 17-year-old girl presenting with seizure H-MRS regarding major and dominant lactate peak at the left hippocampus

**Figure 2:**
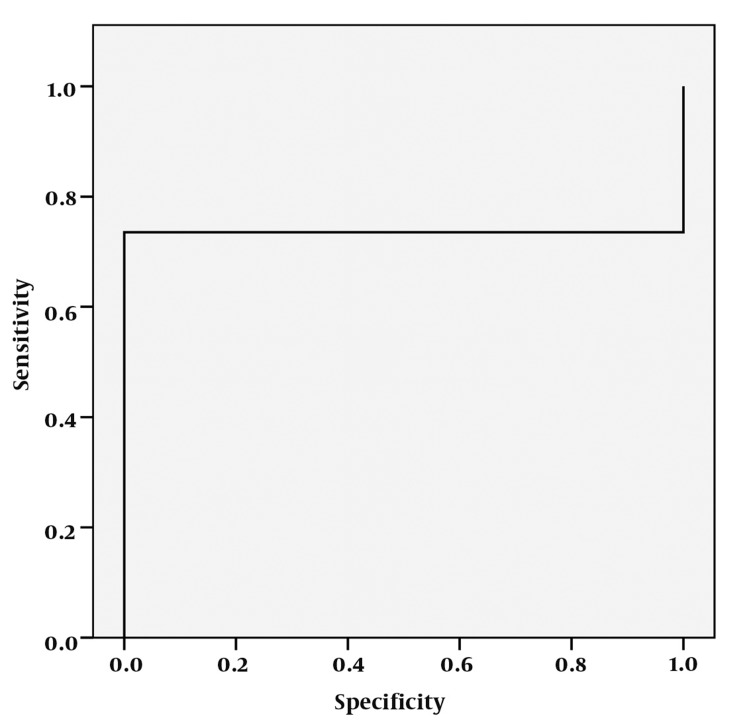
ROC curve analysis for lactate peak

The mean ± standard deviation (SD) of Lac peak was 3.86 ± 2.05 in the right hippocampi and 4.41 ± 2.86 on the left for the patient group. It was almost zero in the control group. According to single-sampling-t-test for both hippocampi, there was statistically significant differences between Lac peaks and the normal presumed values (P < 0.0001 for both hippocampi) (Table 1, Figure 1). In the ROC curve analysis; AUC of 0.735 ± 0.054 was obtained and 2.6625 cut-off value yielded a 73.5% sensitivity and 100% specificity (Figure 2).

**Table 1:** Metabolite (Lac, NAA, Cho, Cre, Glx, MI, Cr2, Cho/Cr, NAA/Cho + Cr, NAA/Cr) Peak Concentrations of the Right and Left Hippocampus, Analysed by t test (P < 0.05)

**Measurement**	**Location**	**No.**	**Mean ± SD**	** Test value = 0**		
				**t**	**SD**	***P* value**
Lac a						
	Right	39	3.86 **± **2.05	11.608	38	< 0.001
	Left	31	4.41 **± **2.68	9.163	30	< 0.001
NAA a						
	Right	39	0.67 **± **0.58	-0.137	38	< 0.001
	Left	31	0.73 **± **0.67	-0.322	30	< 0.001
Cho a						
	Right	39	0.75 **± **0.72	1.895	38	0.066
	Left	31	0.65 **± **0.55	1.266	30	0.215
Cre a						
	Right	39	0.72 **± **0.76	1.799	38	0.080
	Left	31	0.56 **± **0.43	0.724	30	0.474
Glx a						
	Right	39	1.15 **± **1.19	4.646	38	< 0.001
	Left	31	1.16 **± **1.07	4.773	30	< 0.001
MI a						
	Right	39	0.76 **± **0.61	6.137	38	< 0.001
	Left	31	0.60 **± **0.42	5.951	30	< 0.001
Cr2 a						
	Right	39	0.78 **± **0.95	4.219	38	< 0.001
	Left	31	0.54 **± **0.54	4.323	30	< 0.001
Cho/Cr a						
	Right	39	1.44 **± **0.82	4.076	38	< 0.001
	Left	31	1.51 **± **0.77	3.956	30	< 0.001
NAA/Cho + Cr a						
	Right	39	0.71 **± **0.90	-2.011	38	0.052
	Left	31	1.00 **± **1.31	-0.020	30	0.984
NAA/ Cr a						
	Right	38	1.44 **± **1.97	-0.024	38	0.981
	Left	31	2.20 **± **2.59	1.624	30	0.115

^a^ Abbreviations: Cho, Choline; Cho/Cr, Choline/Creatine; Cre, Creatine; Cr2, Phosphocreatine; Glx, Glutamin-Glutamate; Lac, Lactate; MI, Myo-inositol; NAA, N-Asetyl Aspartate; NAA/Cho + Cr, N-Asetyl Aspartate/Choline + Creatine; NAA/Cr, N Asetyl Aspartate/Creatine

**Figure 3:**
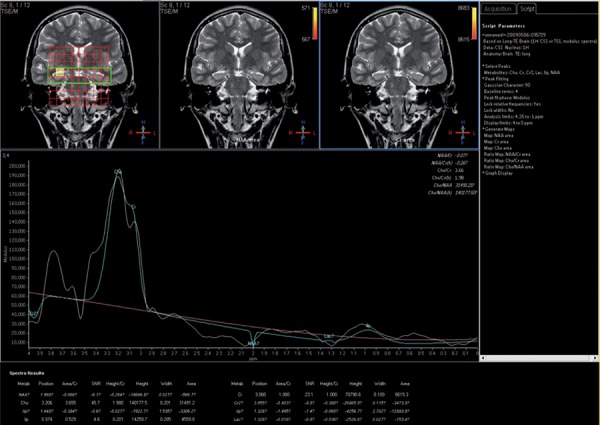
A 19-year-old man with a complaint of headache and a history of seizure MRSI revealed a markedly decreased N-acetyl aspartate peak in the right hippocampus.

The mean ± SD of NAA peaks was 0.67 ± 0.58 in the right hippocampi and 0.73 ± 0.67 on the left for the patient group. This figure was approximately 1.25 in the healthy group. According to NAA peaks for both hippocampi, there was a significant difference from the normal estimated values (P < 0.0001 for both hippocampi) (Table 1, Figure 3). Estimation of AUC and cut-off point for NAA could not be performed and the ROC curve would not be displayed.

**Figure 4:**
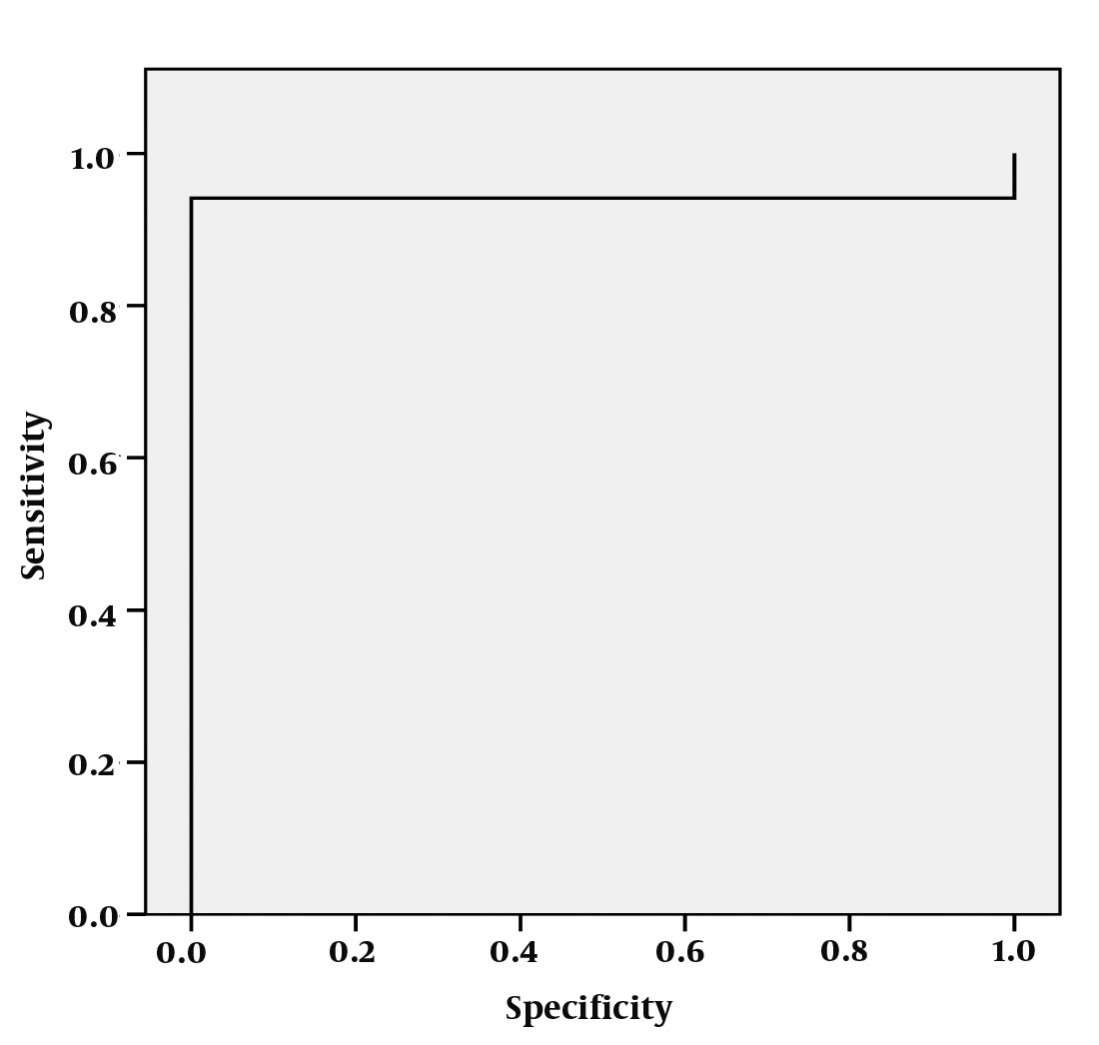
Roc curve analysis for Cho peak

The mean ± SD of Cho peaks was 0.75 ± 0.72 in the right hippocampi and 0.65 ± 0.55 on the left for the patient group. This was about 0.525 in the control group. According to single-sampling-t-test for both hippocampi, there was no statistically significant differences between Cho peaks and the normal calculated Cho values in the control subjects and the healthy opposite hippocampus (P = 0.066 for the right hippocampus and 0.215 for the left) (Table 1). In ROC curve analysis, a AUC of 0.941 ± 0.029 for Cho metabolite was found and a 0.110 cut-off point demonstrated a 94% sensitivity and 100% specificity (Figure 4).

**Figure 5:**
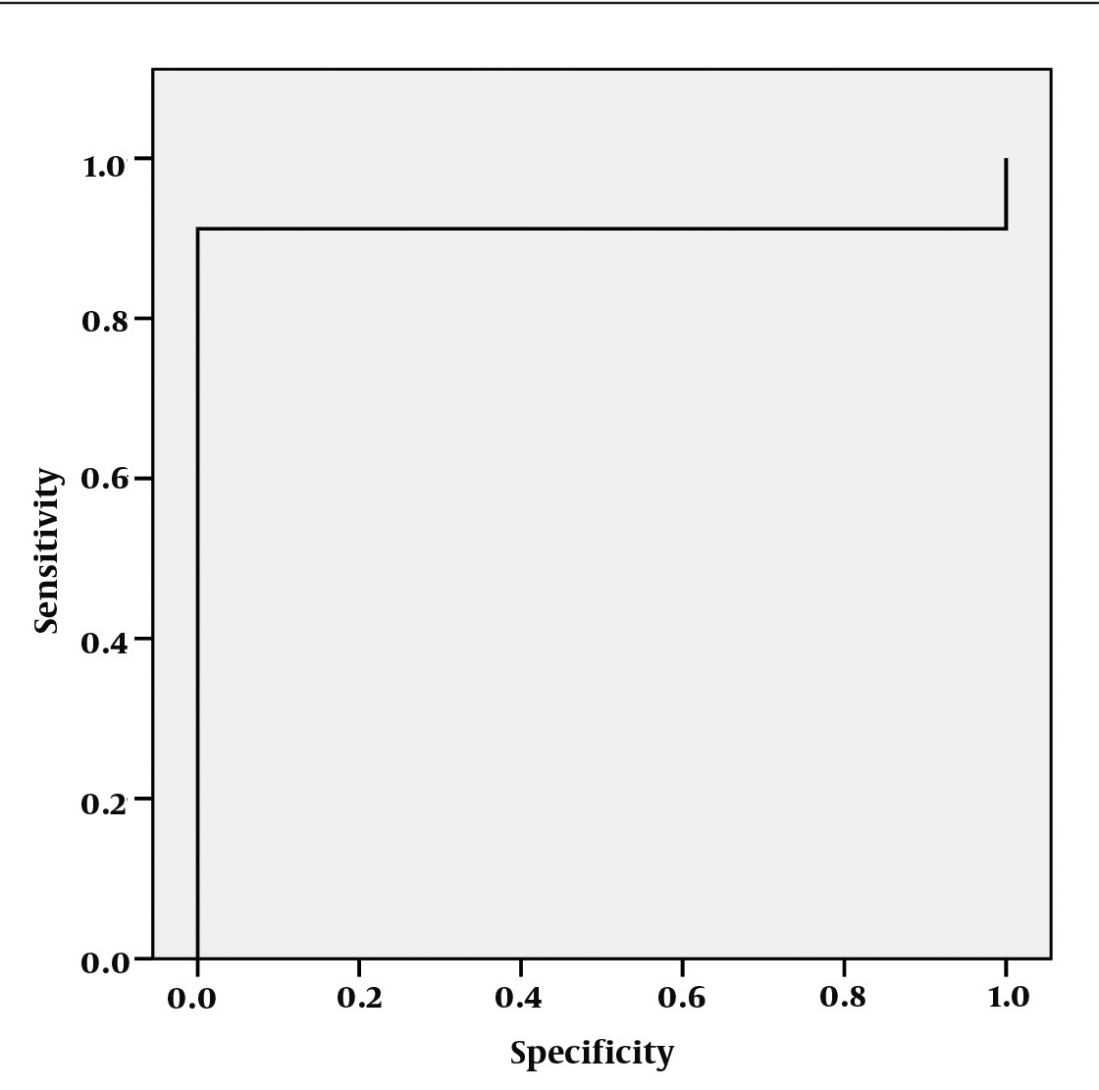
Roc curve analysis for Cre peak

The mean ± SD of Cre peaks was 0.72 ± 0.77 in the right hippocampi and 0.56 ± 0.43 on the left for the patient group. That was about 0.50 for the control group. According to Cre peaks for both hippocampi, there was no statistical difference from the normal Cre values using t-test (P = 0.080 for right hippocampus and 0.474 for the left) (Table 1). An AUC of 0.912 ± 0.032 was shown in the ROC curve and a cut-off value of 0.185 revealed a 91% sensitivity and a 100% specificity (Figure 5).

**Figure 6:**
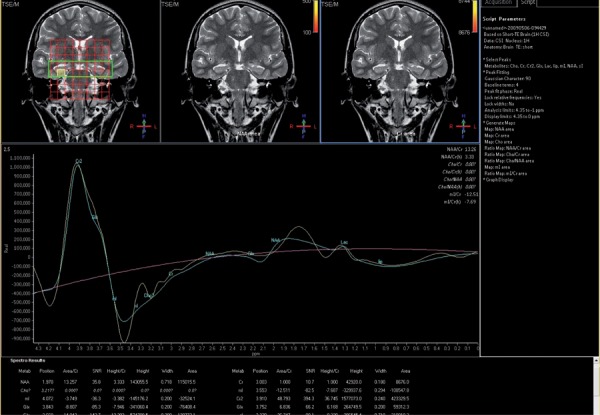
A 22-year-old girl with positive EEG and clinical suspicion for epilepsy H-MRS presented a dominancy of huge glutamate and glutamine peak in the right hipocampus.

**Figure 7:**
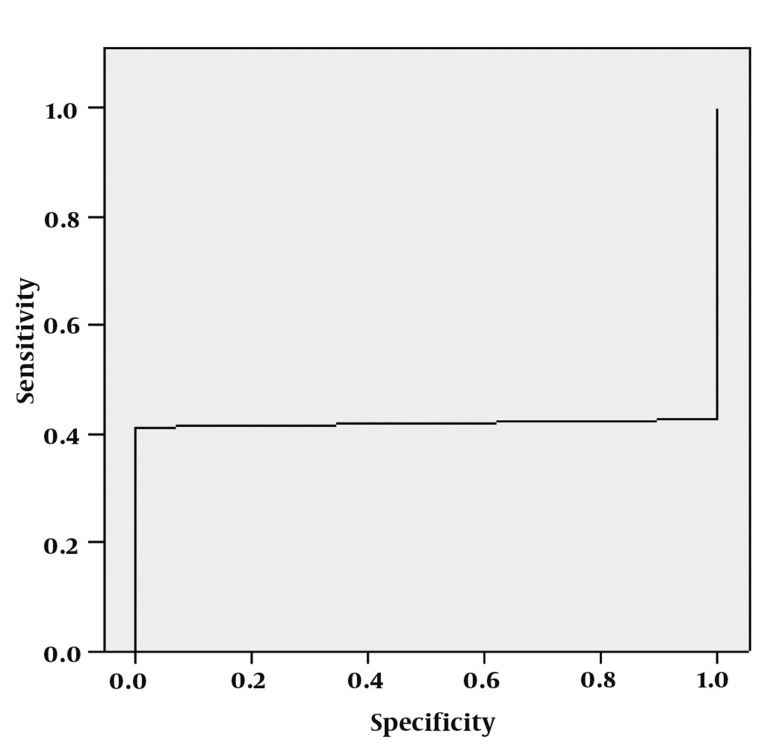
ROC curve analysis for Glx peak

The mean ± SD of Glx peaks was 1.15 ± 1.19 in the right hippocampi and 1.16 ± 1.07 on the left for the patient group and 0.25 for the normal control group. According to single-sampling t-test for both hippocampi, there was a statistically significant difference between Glx peaks and the normal calculated glutamate values (P < 0.0001 for both hippocampi) (Table 1, Figure 6). ROC curve was assessed for Glx with an AUC of 0.450 ± 0.091, but a proper cut-off value could not be fitted (Figure 7).

**Figure 8:**
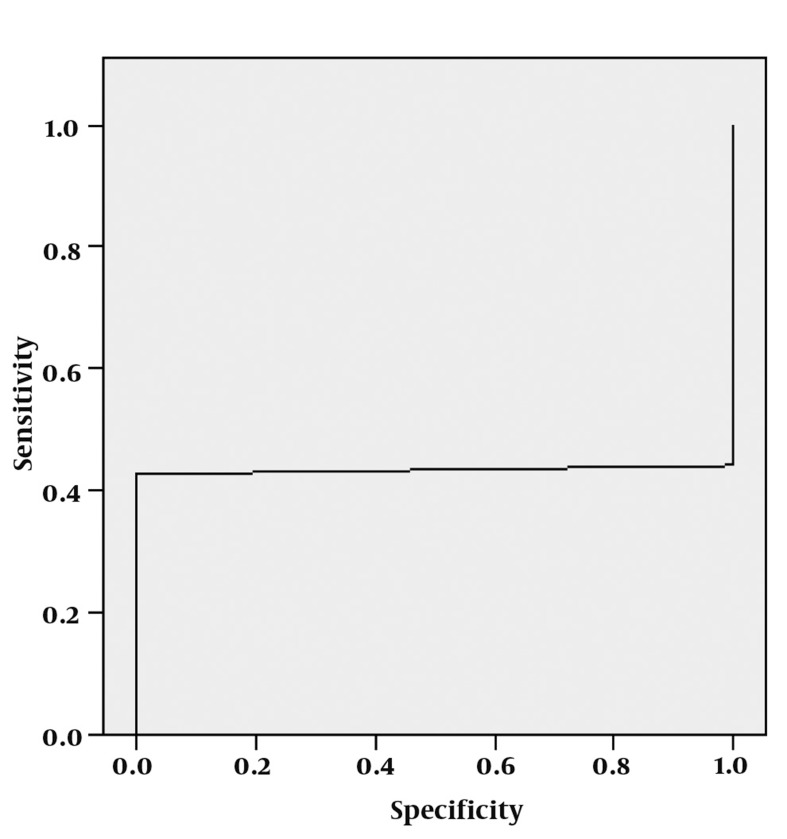
ROC curve analysis for MI peak

The mean ± SD of MI peaks was 0.76 ± 0.61 in the right hippocampi and 0.60 ± 0.42 on the left for the patient group and about 0.15 for the control group. According to the obtained MI values of patients for both hippocampi by using t-test, there was a statistically significant difference from the normal control group values and the healthy opposite hippocampus (P < 0.0001 for both hippocampi) (Table 1), ROC curve was evaluated for MI peak with an AUC of 0.434 ± 0.060, but like the Glx, a proper cut-off value could not be displayed (Figure 8).

**Figure 9:**
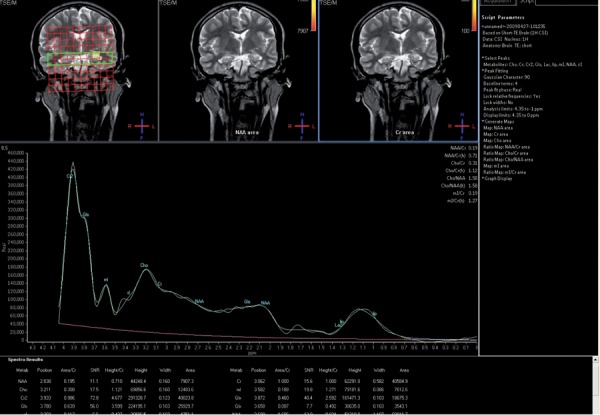
A 40-year-old man with late epilepsy MRSI predicted a dominant phosphocreatine peak in the left hippocampus.

The mean ± SD of Cr2 peaks was 0.78 ± 0.95 in the right hippocampi and 0.54 ± 0.54 on the left for the patients and about 0.125 in the control group. According to Cr2 values for both hippocampi, there was a statistically significant difference between Cr2 peaks and the normal estimated phosphocreatine values (P < 0.0001 for both hippocampi) (Table 1, Figure 9). Estimation of AUC and cut-off point for Cr2 could not be performed and the ROC curve would not be displayed.

The mean ± SD of NAA/Cr, Cho/Cr, NAA/Cho + Cr ratios in the control subjects and healthy hippocampi were 1.45 ± 0.17, 0.60 ± 0.27 and 1.00 ± 0.23, respectively.

**Figure 10:**
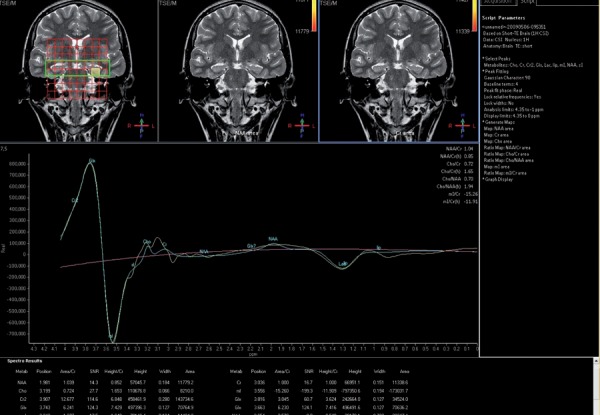
A 30-year-old man with dizziness, mild confusion and a history of epilepsy H-MRS revealed increased Cho/Cre ratio in the left hippocampus.

**Figure 11:**
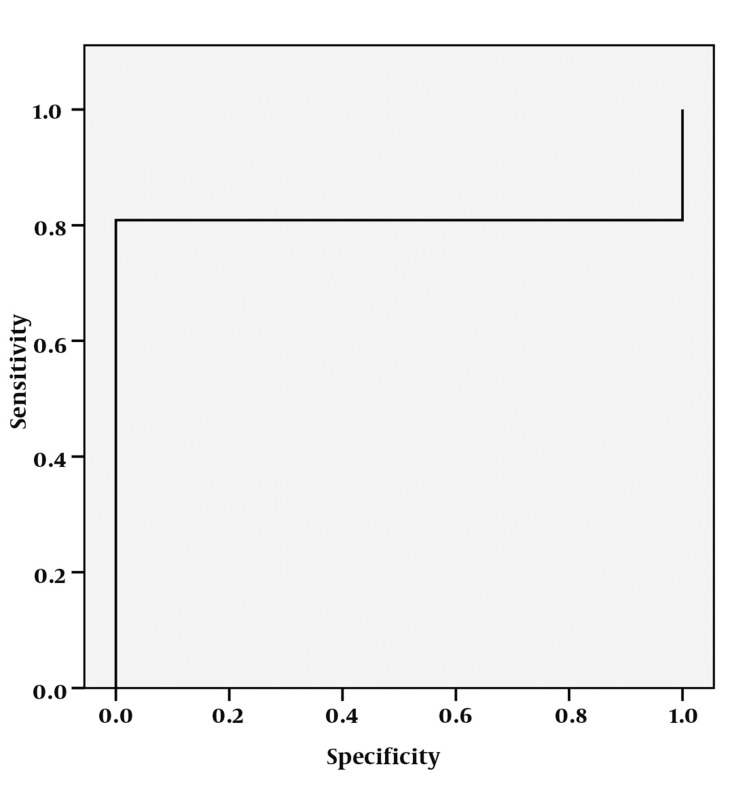
ROC curve analysis for Cho/Cr ratio

The mean ± SD of Cho/Cr ratios were about 1.44 ± 0.85 in the right hippocampi and 1.51 ± 0.77 on the left for the patient group and about 0.60 in the normal healthy group. According to single-sampling t-test for both hippocampi, there was statistically significant differences for Cho/Cr ratio between patients and the normal ones (P < 0.0001 for both hippocampi) (Table 1, Figure 10). In the ROC curve analysis, an AUC of 0.809 ± 0.048 was determined and a 0.560 cut-off value showed an 80% sensitivity and 100% specificity to reveal epileptic hippocampus clearly (Figure 11).

The mean ± SD of NAA/Cho + Cr ratios was 0.71 ± 0.90 in the right hippocampi and 1.00 ± 1.31 on the left for the patients. That ratio was about 1 in the normal control group. According to NAA/Cho + Cr ratios for both hippocampi, there was no statistically significant differences from the normal control subjects and healthy hippocampi (P = 0.052 for right hippocampus and 0.984 for left) (Table 1).

**Figure 12:**
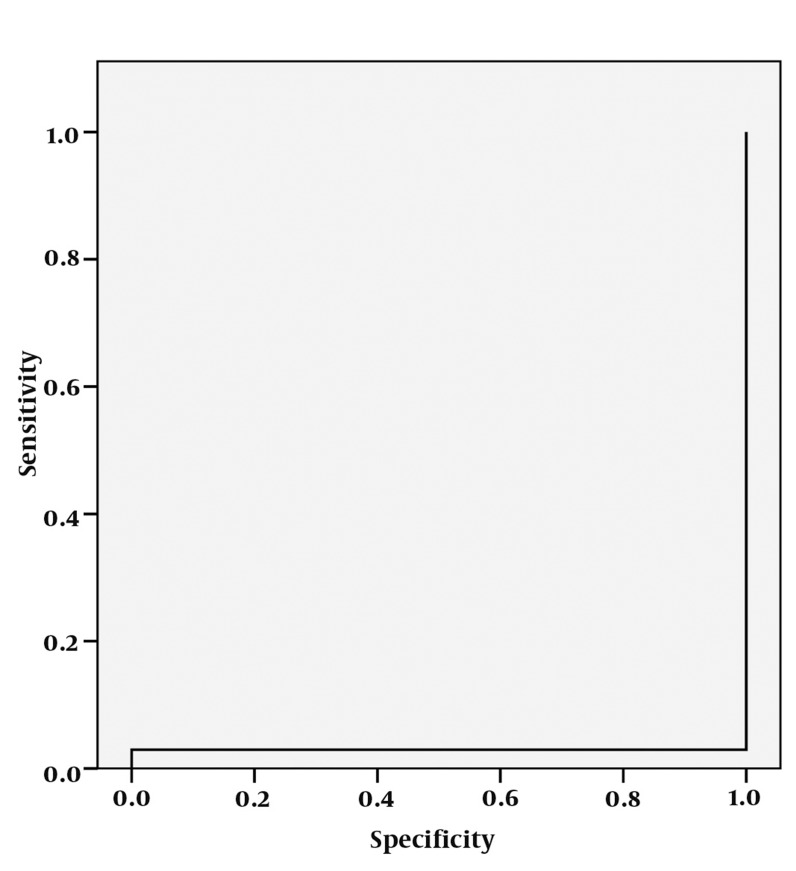
ROC curve analysis for NAA/Cr ratio

The mean ± SD of NAA/Cr ratios was about 1.44 ± 1.97 in the right hippocampi and 2.20 ± 2.59 on the left for the patient group. It was about 1.45 in the control subjects. According to single-sampling t-test for both hippocampi, there was no statistically significant differences for NAA/ Cr ratio between epileptic and the normal healthy hippocampi (P = 0.981 for the right hippocampus and 0.115 for the left) (Table 1). The ROC curve analysis yielded an AUC of 0.029 ± 0.020 for NAA/Cr ratio and this analysis could not fit a proper cut-off value with higher sensitivity and specificity for detecting the abnormal epileptic hippocampus (Figure 12).

For the bilateral hippocampal involvement in 22 patients, paired-t-test was applied and only MI metabolite had statistically significant differences between the left and the right hippocampus (P = 0.032). MI metabolite concentration was about 0.89 ± 0.66 in the right and 0.60 ± 0.47 in the left hippocampus. The other metabolites (NAA-Glx-Cho-Cre-Lac) had no statistically significant differences with regard to both hippocampal yields with P values ranging from 0.234-0.984, NAA/Cr, NAA/Cho + Cr and Cho/Cr did not reveal any statistically significant differences between the right and left hippocampi either,with P values of 0.124 for NAA/Cre, 0.800 for Cho/Cre and 0.207 for NAA/Cho + Cre.

The sensitivity of MRSI for lateralization of epileptic foci in all 70 hippocampi was 96% and the specificity was 50% (Table 2). In the left hippocampi, MRSI detected all the lateralized epileptic activity with regard to EEG results. On the right side, four false negative output were found using MRSI according to EEG findings; therefore, in the right hippocampi the sensitivity was a little lower (92%) (Table 2).

**Table 2:** Sensitivitiy and Specificity of MRS

**Location**	**MRS **a** Negative + EEG a Negative**	**MRS Negative + EEG Positive**	**MRS Positive + EEG Negative**	**MRS Positive + EEG Positive**	**Total**
Both	1 (25)	3 (75)	1 (2)	65 (98)	70 (100)
Right	1 (25)	3 (75)	0 (0)	35 (100)	39 (100)
Left	0 (0)	0 (0)	1 (0)	29 (100)	31 (100)

^a^ Abbreviation: EEG, electro-encephalography; MRS, proton MR spectorscopy

## 5. Discussion

The new imaging techniques over the past 20 years have enabled the non-invasive analysis of abnormalities, preceding epileptic seizures and also allowed the use of these approaches in order to reveal the etiologic mechanisms of epilepsy [1][3]. Many diagnostic imaging tools such as brain MRI, PET, SPECT and H-MRS may be administered and correlated with the results of classical EEG and used in the analysis of epilepsy [1][5][7]. MRI and H-MRS offer a lot of qualitative and quantitative data which can help to localize the epileptic lesions and provide an insight into the biophysical and biochemical processes related to epileptic seizures [1][4][5][6]. The early reports for H-MRS in patients with MTLE were published in the 1990s which tried to lateralize the focal epileptic foci [1][7][9][12][13][14]. Examination of patients with epilepsy using MRSI is now focused on the observation of changes in NAA, Cr, Cr2, Cho, Glx and GABA (Gama-aminobutyric acid) signals, their correlations with the results of MRI, EEG findings and the clinical features [1][3][5][6][7][10][11]. In this paper, H-MRS was discussed as a method for lateralization or localization of the epileptogenic zone in MTLE. It was the best-recognized surgically proven epilepsy syndrome; the epileptogenic zone consisted of the amygdalo-hippocampal complex [1][3]-[13]. Approximately, 60% of MTLE patients, were predicted with severe unilateral hippocampal atrophy and a typical macroscopic feature of hippocampal sclerosis, also called Ammon’s horn sclerosis or mesial temporal sclerosis (MTS); ‘’Hippocampi of these patients revealed astrogliosis with prominent neuronal cell losses histopathologically’’ [1][5][10][14].

MRI and at the same time H-MRS, which are non-invasive techniques, play an extremely important role in the diagnosis of hippocampal sclerosis and MTLE; MRI shows an atrophic-small or firm hippocampus and also hyperintense, diffuse edematous areas at the hippocampi on T2W images [1][4][6][8][13]. H-MRS predicted the metabolite peaks, measured in different parts of both hippocampi and NAA decrease in the spectrum usually provides the lateralization and localization of the epileptic focus [1][5][6][7][8][10][12][13][14]. As mentioned above, the most important signal in the MRSI spectra is NAA which describes the neuronal loss and dysfunction. NAA is synthesized in the mitochondria of neurons from aspartate and acetyl-CoA and its synthesis is controlled by aspartate-N-acetyltransferase [1][5][14][15]. The other important metabolites, especially for the treatment of epilepsy are Glx and GABA, of which Glx is the main excitatory neurotransmitter and GABA is the main inhibitory neurotransmitter [1][3]. With the impaired function of GABA, Glx becomes higher leading to hyperexcitability and spontaneous epileptic activity; therefore, administration of GABAergic anti-epileptic drugs can easily improve and control the seizures [1][3][15]. Previous H-MRS studies have shown elevation of Glx in patients with MTLE without the evidence of hippocampal sclerosis on MRI [3][16].

NAA appears to be sensitive to neuronal loss and is often reduced in the region of sclerotic hippocampi and MTLE [3][6][7][10][11][12][13][14], “may be significantly reduced in the regions distant from the epileptogenic foci mainly in the multilobar and the extra-temporal lobe epilepsies’’ [10] and also particularly reduced in the contralateral normal appearing hippocampi [12][13][14][16]. Simister et al. [16], Hammen et al. [6], Achten et al. [12] and Ende et al. [13] thought that a decreased NAA peak would be the marker of epileptic foci in the diseased hippocampus and a clue for the contralateral hippocampus involvement. Kuzniecky et al. [17] and Vielhaber et al. [5] revealed that decreased NAA peak in MTLE, especially in the hippocampal sclerosis-Ammon’s horn sclerosis might be due to the impaired mitochondrial function, not correlated with the neuronal cell losses. In our study, NAA peak was significantly decreased in the epileptic hippocampi similar to the previous studies, but we did not evaluate the normal appearing contralateral hippocampi in the EEG and to our belief, loss of hippocampal NAA in MTLE was also due to the neuronal cell loss and gliosis.

According to Cho and Cr peaks; there was no general concept in the literature, Simister et al. [3], Simister et al. [16], Hammen et al. [6] and Achten et al. [12] thought that both metabolites increase in MTLE and Thompson et al. [7], Ende et al. [13] and Kuzniecky et al. [17] declared that there even was reduction of both metabolites especially in chronic sclerotic hippocampi. In our study; there was no significant statistical change between the seizured hippocampi and normal contralateral hippocampi.

Simister et al. [3] and Hammen et al. [6] both reported an obvious increasing trend in Cr plus Cr2 due to gliosis in mesial temporal structures and they also showed that the levels fell immediately after the improvement of the seizures. Our Cr2 peak was also high in the epileptogenic focus similar to these mentioned studies. Petroff et al. [15], McLean et al. [18] and Simister et al. [3][16] declared that ‘’glutamate and glutamine (Glx) were the main metabolites beneath the NAA concentration and it was significantly elevated in the diseased and sclerotic hippocampi’’, Glx peak was also high in our MTLE series much the same as their studies. Mc Lean et al. [18] and Simister et al. [3] showed a precisely high MI peak due to gliosis of hippocampi in their MTLE series and thought that this was because of the myo-inositol predominance in glial cells. In our study, we had similar results; the MI peak was significantly elevated.

Elevated Lac levels in the MTLE patients were shown in very few studies, Thompson et al. [7] and Vielhaber et al.

[5] reported a small Lac increase in the post-ictal MR spectras of their patients. They also suggested that Lac might persist only a short time after the controlled seizure and its peak should be due to the activated glycolytic activity and mitochondrial dysfunction. In our study, which was just controversial, Lac was the dominant metabolite in the hippocampi of MTLE patients and its peak was extremely high in the damaged mesial temporal structures; therefore, we thought that Lac was an additional marker for seizure activity of MTLE patients.

In previous studies, most of the authors explained and dedicated on the importance of significantly decreased NAA/Cr and NAA/Cho + Cr ratios of the epileptic foci in the temporal lobes. They believed that “both ratios were strongly correlated with the lateralization and localization of the epileptogenic area, degree of seizures and epileptic discharges’’ [4][6][7][8][10][11][12][13][16]. Some authors also believed that even after the anterior temporal lobe resection, normalization of both ratios may take a great amount of time and needed to be validated by strict follow up [4][6][16]. Li et al. [10] showed that decreased NAA/ Cr ratio indicating recent neuronal injury was often widespread in either temporal and/or extra-temporal epilepsy. Some authors also found out that both ratios would also be decreased in the contralateral temporal lobe [4][8][13]. Cohen-Gadol et al. [4], Li et al. [8], Hammen et al. [11] and Simister et al. [16] performed the diagnosis and lateralization of the epileptic activity with regard to decreased NAA/Cr ratio. In our series, we did not have any significant statistical differences between the epileptic hippocampi and the normal appearing ones, with regard to the NAA/Cr and NAA/Cho + Cr ratios, but we had precisely declined NAA/Cr and NAA/Cho + Cr ratios due to the depressed NAA metabolite level and slightly increased Cho and Cre peaks.

Thompson et al. [7] and Achten et al. [12] tried to focus on the epileptic zone using Cho/Cr ratio, but as both metabolites are in the similar trend, increasing or decreasing, they performed worser than NAA/Cr plus NAA/Cho + Cr ratios and therefore, both authors thought that Cho/ Cr ratio has no role in the lateralization of MTLE. In our study, Cho/Cr ratio in the diseased hippocampi showed significant alteration from the normal hippocampi, but to our knowledge, only with this ratio localization of the epileptic activity could not be performed properly.

As a summary, in our 46 patients with MTLE in 70 hippocampi, there were significantly elevated Lac, MI, Glx, Cr2 and decreased NAA peaks and at the same time elevated Cho/Cr ratios and declined NAA/Cr and NAA/Cho + Cr ratios in comparison to control subjects and the normal contralateral hippocampi. We thought that alterations of these metabolites seen in MRSI should be enough for the correct lateralization and localization of epileptic activity in the mesial temporal lobe structures. Beneath the known variance of some unique metabolites in the H-MRS, an unknown and surprising Lac dominancy in the MR spectra of MTLE could also be used for an extra-marker in the diagnosis of seizure activity.

Our findings demonstrate that H-MRS can be applied as an additional method in the diagnosis of MTLE. Patients with MTLE were correctly lateralized with MR-Spectroscopy when compared with EEG findings and clinical features. Although in our study the specificity of MRSI was 50% and seems to be low, with a 96% sensitivity, HMRS may be a helpful and non-invasive technique that may present the abnormal hippocampus with partial seizures. The previous studies in the literature and our results present that MRSI can easily be considered as an alternative modality of choice in the diagnosis of temporal lobe epilepsy due to its higher sensitivity compared to other imaging modalities for the detection of epileptic metabolites in mesial lobe structures and with further increasing experiences mainly in molecular imaging, HMRS may become the most important technique used in epilepsy treatment centers in the future.
